# 

**Published:** 1913-09-06

**Authors:** 


### Personalia.

DR. FRANCIS WARNER has been elected consulting physician to the London Hospital. Dr. Warner has for many years been the physician in charge of the health of the nurses. This duty Dr. Thompson has now agreed to take up.

SIR HENRY LUCY (Toby, M.P., of *Punch*) has given £1,000 to the London Hospital for the naming of a bed, with a special wish that so far as possible it should be used for the benefit of the relatives of poorer members of the Press Gallery.

MRS. WYLLIE, who has reported her intention to invest £10,000 for the widows and orphans of fishermen of the district between Ferryden and Bervie, Montrose, had given previously £5,000 to the Montrose Royal Infirmary and £1,500 to endow beds at the Edzell Convalescent Home.

IN order to fill the vacancy among the physicians at the London Hospital, caused by the retirement of Dr. Warner, Dr. Henry Head, F.R.S., the senior of the assistant physicians, has been appointed to this post. The vacancy in the post of assistant physician has been filled by the appointment of Dr. Charles Miller.

